# Effects of Immersion Freezing on Ice Crystal Formation and the Protein Properties of Snakehead (*Channa argus*)

**DOI:** 10.3390/foods9040411

**Published:** 2020-04-02

**Authors:** Shulai Liu, Xiaohong Zeng, Zhenyu Zhang, Guanyu Long, Fei Lyu, Yanping Cai, Jianhua Liu, Yuting Ding

**Affiliations:** 1 College of Food Science and Technology, Zhejiang University of Technology, Chaowang Rd 18, Hangzhou 310014, China; slliu@zjut.edu.cn (S.L.); 15280857911@163.com (X.Z.); zzy18058789757@163.com (Z.Z.); lgy_1999@163.com (G.L.); lufei@zjut.edu.cn (F.L.); caiyp@zjut.edu.cn (Y.C.); jhliu@zjut.edu.cn (J.L.); 2 National R & D Branch Center for Pelagic Aquatic Products Processing (Hangzhou), Hangzhou 310014, China; 3 Institute of Ocean Research, Zhejiang University of Technology, Jiashan Rd 33, Hangzhou 310032, China; 4 Collaborative Innovation Center of Seafood Deep Processing, Dalian Polytechnic University, No. 1st Qinggongyuan, Dalian 116034, China

**Keywords:** immersion freezing, snakehead, ice crystal, microscopic analysis, protein properties

## Abstract

This study aimed to evaluate the effect of immersion freezing (IF) at different temperatures on ice crystal formation and protein properties in fish muscle. Snakehead blocks were frozen by IF at −20, −30, and −40 °C, and conventional air freezing (AF) at −20 °C. The size of ice crystals in the frozen samples was evaluated using Image J software. Changes in protein properties were analyzed by Fourier transform infrared spectroscopy (FT-IR) and differential scanning calorimetry (DSC). Snakehead blocks frozen using IF contained smaller ice crystals and better microstructures, especially at lower temperatures. The mean cross-sectional areas of ice crystals formed in the frozen samples were 308.8, 142.4, and 86.5 μm^2^ for IF treatments at −20, −30, and −40 °C, respectively, and 939.6 μm^2^ for the AF treatment. The FT-IR results show that protein aggregation in the frozen fish blocks was manifested by a decrease in α-helices connected to the increased random coil fraction. The DSC results show that samples prepared by IF had a higher denaturation enthalpy (*∆H*) and denaturation maximum temperature (*Tmax*) than those prepared by AF. These results confirm that IF generated a larger number of smaller ice crystals, which is conducive to food preservation.

## 1. Introduction

Freezing is among the most important methods for long-term food preservation because it can retain the original flavor, color, and nutritional value of food [1]. The formation of intercellular ice crystals affects the quality of frozen products, with the freezing rate being known to influence the size, number, and distribution of ice crystals formed during freezing [1,2,3,4]. Large and irregularly distributed crystals are the main factors that diminish frozen food quality, while small and uniformly distributed crystals can cause less damage to cell tissues and protein structure [5]. In the food industry, air-blast, plate contact, and fluidized-bed freezing are the most common methods used for rapid freezing. However, the high freezing rates can also lead to a lower food value [6]. For example, cryogenic freezing generates intensive nucleation and large amounts of intracellular ice crystals, but may cause mechanical damage to frozen foods [7]. Therefore, new freezing technologies continue to be proposed and developed.

Immersion freezing (IF) uses a liquid coolant as the heat-transfer medium, with the food either directly or indirectly contacting the refrigerant, resulting in rapid cooling and freezing due to the high thermal coefficient of the liquid medium [8]. In general, liquids have a thermal conductivity of 0.116–0.628 w/(m·k) and air has a thermal conductivity of 0.024 w/(m·k), with the former leading to a higher freezing rate, uniform nucleation in foods, and the formation of smaller ice crystals. As a rapid freezing technology, IF creates a large amount of small ice crystals, which helps improve the quality of frozen foods [9].

Recently, IF has received attention from many food researchers, mainly due to its potential for improving frozen food quality. Several studies have been conducted on the kinetics of IF and the characteristics of ice crystals formed in foods such as fruits (strawberry and apple) [10,11], vegetables (carrot and potato) [12,13], pork [14], and large pelagic fish (yellowfin tuna and skipjack tuna) [15]. These studies have generally demonstrated that IF treatment has a high freezing rate, which helps preserve the original food quality. From a microstructural perspective, damage to cells is minimized in IF-treated samples and the protein structural stability is maintained owing to the small ice crystal size, resulting in a significant improvement in product quality [16].

However, further studies are required, for example, to understand the protein properties of aquatic food products subjected to IF. Snakehead (*Channa argus*) is a fish belonging to the *Channidae* family that is widely cultivated in southern and southeastern Asia [17]. Snakehead is rich in protein and amino acids, and has become increasingly popular among consumers. However, few studies have investigated the effects of IF on snakehead muscle. Therefore, this work aimed to determine changes in ice crystal formation and protein properties in snakehead during different freezing processes. Accordingly, IF was performed at three different temperatures (−20, −30, and −40 °C). Furthermore, Fourier transform infrared spectroscopy (FT-IR) and differential scanning calorimetry (DSC) were used to analyze changes in the secondary structure and thermal stability of proteins. These results were analyzed in comparison to samples prepared by conventional air freezing.

## 2. Materials and Methods

### 2.1. Sample Preparation

Fresh snakehead (*Channa argus*, 1.5 ± 0.2 kg) was purchased from local suppliers (Hangzhou, China). After removing the head and tail, and cleaning each fish, samples were prepared by cutting along the muscle fiber using a sharp cylindrical tool and surgical blade, and then slicing into 2 × 2 × 2 cm blocks. Each fish block was wrapped in polyethylene (PE) film to avoid penetration during the freezing processes.

### 2.2. Freezing and Storage

The snakehead blocks were divided into two groups (A and B). Group A was used for IF treatments at −20, −30, and −40 °C. The multicomponent refrigerant liquid used for IF was composed of 24% ethanol, 20% propylene, and 7% NaCl (approximately freezing point, −48.8 °C). A K-type thermocouple digital thermometer (diameter, 0.5 mm; accuracy, ± 0.1 °C; Omega Corp, Stamford, CT) was inserted into the geometric center of one of the samples to record the temperature variation. The freezing processes were stopped when the temperature at the sample center reached −18 °C. Group B was subjected to conventional air freezing at −20 °C until the center temperature reached −18 °C, measured as described for Group A.

Once the freezing process was complete, the frozen samples were temporarily stored in a conventional freezer (−20 °C) until further treatment.

### 2.3. Microscopic Analysis

From the frozen stored snakehead block (−20 °C), two pieces (approximately 5 mm in length) were cut from the center, transversally to the muscle fiber, using a blade previously cooled to −20 °C. An indirect method, known as isothermal freezing substitution, was used to observe spaces produced by ice crystals in the fish tissue, similar to that reported in a previous study of ice crystal formation in salmon [18]. The frozen snakehead blocks were embedded with an embedding agent (O.C.T. Compound, Japan), placed in a low temperature environment until the embedding agent had solidified, and then sliced into sections. Using a semi-conductor frozen slicer (Cryostar N × 50, Thermo Electron Corp., USA), the embedded samples were sliced into 7-μm-thick specimens along the direction of the muscle fibers, and hematoxylin-eosin staining was used to observe ice crystals.

All prepared slides were observed using a light microscope (Olympus B × 41, Japan) equipped with a camera (Olympus Corp., Japan). Images of the slides were treated using Image J software (National Institutes of Health, USA). Three parameters, namely the cross-sectional area, equivalent diameter, and roundness, were used for microscopic analysis. The roundness (R) was calculated as follows:

(1) $$R=\frac{4\pi A}{P^{2}}$$

where A is the cross-sectional area and P is the perimeter (the length of the outside boundary of the observed object). For each case, more than 100 frames (ice crystals or muscle fibers) were evaluated.

### 2.4. Fourier Transform Infrared Spectroscopy (FT-IR)

Myofibrillar protein (MP) is the major protein in fish and is mainly composed of myosin and actin. MP was extracted from snakehead using the modified method from Pan et al. [19]. Frozen fish muscle (1.0 g) was cut up and homogenized with 20 mL 0.05 mol/L NaCl. Each 20 s of homogenization was followed by a 20-s rest interval to avoid overheating during extraction. Then, the sample was placed in fridge at 4 °C for 30 min, and centrifuged at 10,000 r/min for 20 min at 4 °C. The above process was repeated once. The precipitate was collected and homogenized with chilled 0.6 mol/L NaCl for 1 min. Then, the sample was placed in a fridge at 4 °C for 90 min, and centrifuged at 10,000 r/min for 20 min at 4 °C. A pellet of MP was collected by centrifuging at 10,000 r/min for 10 min at 4 °C and dissolved by chilled 0.6 mol/L KCl. Chilled (4 °C) deionized water (40 mL) was added to 10 mL of supernant to precipitate MP. Undissolved material was removed from the preparation by centrifuging at 10,000 r/min for 10 min at 4 °C. MP were frozen at −20 °C for 24 h and then placed in a freeze-drier (Zirbus, Germany) for 48 h at −50 °C. The dried samples were ground into fine powder with a mortar and pestle [20].

The FT-IR spectra of freeze-dried MP from snakehead were obtained using an FT-IR spectrometer (Nicolet 6700, Thermo Electron Corp., USA) equipped with an interferometer. This method was based on a literature procedure with a slight modification [21]. FT-IR samples were prepared by mixing freeze-dried MP powder (2 mg) and KBr (100 mg), which were then ground uniformly. The samples were then scanned in transmission mode using a scan range of 4000–400 cm^−1^, with a total of 32 scans performed at a 4-cm^−1^ resolution, and were averaged [22].

All spectra were preprocessed using Omnic 8.0 software (Thermo Fisher scientific Inc. Waltham, MA). The raw absorbance spectra were smoothed using nine-point Savitzky-Golay smoothing functions. Gaussian curve fitting of the deconvoluted amide I band (1600–1700 cm^−1^) was performed using Peakfit 4.12 software (SeaSolve Software Inc., San Jose, CA, USA).

### 2.5. Differential Scanning Calorimetry (DSC)

Different proteins have different phase transition peaks and thermal denaturation temperatures. Fish proteins include myosin, sarcoplasmic, and actin [23]. Differential scanning calorimetry (Model Q200; TA Instruments, New Castle, DE, USA) can be used to analyze the different thermal properties of proteins and the degree of denaturation in fish samples after different treatments. About 10 mg of thawed sample was placed in a chrome-resistant crucible and compressed, with an empty aluminum crucible used as the control. The temperature-control procedure was conducted at a rate of 10 °C/min from 10 to 90 °C. The denaturation enthalpy (*∆H*) and denaturation maximum temperature (*Tmax*) were calculated using TA Universal Analysis software (TA Instruments, USA). Three replicates were performed for each sample.

### 2.6. Statistical Analysis

The data are expressed as means ± standard deviation. Origin 8.0 software was used for data processing and diagram construction. The data analysis was performed using SPSS 20.0 software (IBM, Chicago, USA). One-way analysis of variance (ANOVA) followed by Turkey procedure was used to determine the significance (*p* < 0.05) of the difference between means.

## 3. Results and Discussion

### 3.1. Thermal Characteristics in IF Processes

**Figure 1:**
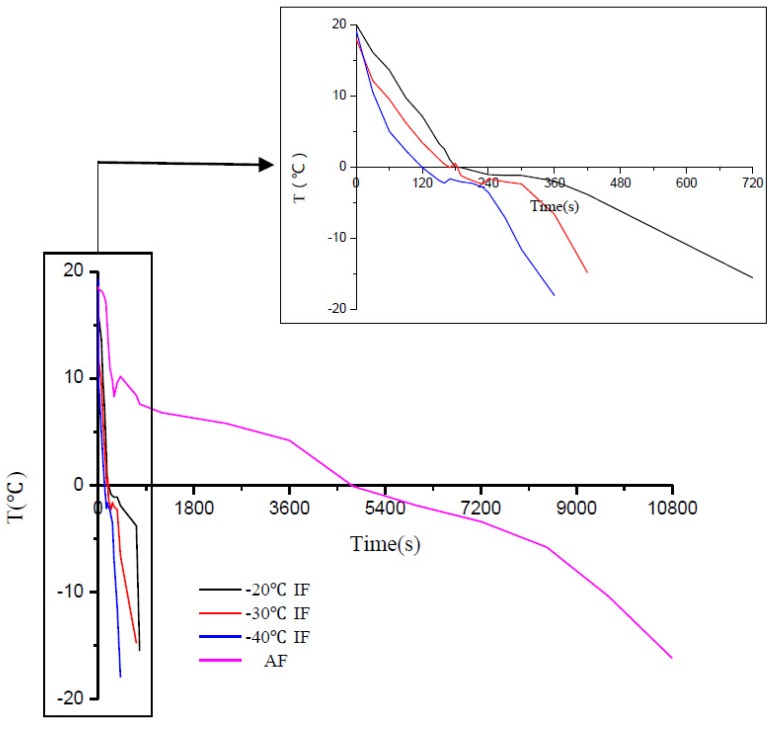
Freezing curves for the centers of snakehead blocks under different freezing conditions.

Changes in the temperature at the center of the snakehead blocks were recorded during each freezing process (Table 1 and Figure 1). Freezing curves have been reported as useful tools for indicating the characteristics of a freezing process [24]. Figure 1 shows freezing curves for the snakehead blocks under different freezing conditions. These curves showed that the rate of temperature decrease during IF was significantly higher than during AF, with a lower IF temperature leading to a faster decrease in temperature. The IF process took significantly less time to freeze the samples than AF. However, different freezing treatments can affect the freezing temperature of the sample center [11,25]. Compared to AF samples, a significant freezing point depression was observed in IF samples. The snakehead block freezing points were about −2.0, −2.3, and −2.4 °C for IF treatment at −20, −30, and −40 °C, respectively, and −1.6 °C for AF treatment. These different sample center freezing temperatures were mainly due to the use of different media (air and liquid) and their different heat transfer efficiencies. The heat transfer efficiency of a liquid is more than 10 times that of air, which affects the rate of crystal nucleation and the release of latent heat during freezing.

The time taken for each freezing stage observed for IF at three different temperatures (−20, −30, and −40 °C) and the AF treatment are shown in Table 1. Significant differences were observed among the total freezing times (time taken from the initial temperature of 4 °C to reach −18 °C). AF had the longest freezing time (112 min) with the lowest freezing rate. IF at −40 °C had the shortest freezing time (4.4 min), followed by IF at −30 °C (6.5 min) and −20 °C (11.3 min). Moreover, significant differences were observed among the samples treated at three different IF temperatures (*p* < 0.05).

In general, the total freezing process can be divided into three stages [2]: precooling (4 to 0 °C), phase change (0 to −5 °C), and subcooling (−5 to −18 °C). AF and IF showed significant differences at each stage (*p* < 0.05), but there were no significant differences (*p* > 0.05) between the precooling and subcooling stages for IF at −30 °C and −40 °C (Table 1). The phase change stage, also called the zone of maximum ice crystallization, is the most important stage in the freezing process [26,27]. During this stage, nucleation and subsequent crystal growth takes place. Minimizing the time taken for the phase change stage contributes to the formation of small and regular ice crystals and improves the quality of frozen food [13,28]. The length of the phase change stage was significantly different (*p* < 0.05) among the four different freezing treatments and was longer than the other two stages (Table 1). Compared to the AF treatment, IF reduced the length of the phase change stage by more than 90%. Therefore, IF treatment is a promising technique for preserving the quality of frozen food and will be advantageous for use in the frozen food industry [16].

### 3.2. Ice crystal Microstructure

**Figure 2:**
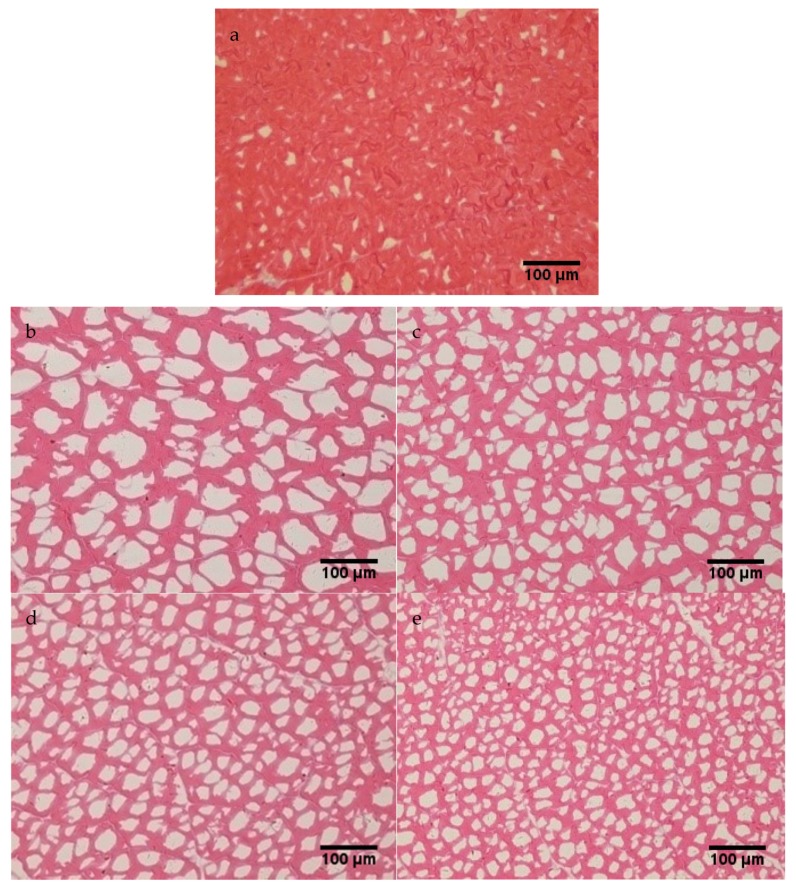
Micrograph images of transversal cuts of muscle samples treated under different freezing conditions: (**a**) the fresh sample, (**b**) −20 °C AF, (**c**) −20 °C IF, (**d**) −30 °C IF, and (**e**) −40 °C IF.

Microstructure integrity is an important factor in determining the quality of frozen food and mainly depends on the size and distribution of ice crystals formed during freezing [29]. Micrograph images of transversal cuts of muscle samples frozen under different freezing conditions are shown in Figure 2. The muscle fibers were stained red, with white zones representing areas left by the ice crystals. The size and location of the ice crystals are greatly dependent on the freezing time and freezing rate, with a shorter freezing time producing smaller ice crystals [30]. Compared to the fresh sample (Figure 2a), the frozen samples showed a different level of tissue shrinkage during the histological procedure. Visual inspection of the micrographs showed that different freezing conditions resulted in different ice crystal sizes and distributions. The ice crystals produced by AF (Figure 2b) were markedly larger than those produced by IF (Figure 2c–e). The range and size of the ice crystals in AF-treated and IF-treated samples were determined to be in the order b > c > d > e (see Figure 2), which implied that the freezing rate was critical to the formation and growth of ice crystal [3]. Furthermore, it can be assumed that the AF process (Figure 2b) caused greater damage to the fish texture than the IF process (Figure 2c–e).

AF at −20 °C exhibited slow freezing, which created larger sized and irregularly shaped ice crystals (Figure 2b). The cross-sectional area of the AF ice crystals was 939.6 ± 134.2 μm^2^, which was larger than those produced by the IF treatments (Table 2). This illustrated that a low rate of nucleation resulted in large and mainly extracellular ice crystals [9,31]. Therefore, slow freezing methods, such as AF, usually cause serious damage to the cell structure and reduce the quality of frozen foods.

IF had a rapid freezing rate and formed ice crystals that were smaller and more uniform than those afforded by AF. Among the three IF treatments, the lowest temperature (−40 °C) produced significantly (*p* < 0.05) smaller ice crystals and a more regular distribution (Figure 2c,d). The cross-sectional areas of the ice crystals were 408.8 ± 76.8, 242.4 ± 47.0, and 76.5 ± 6.4 μm^2^ for samples subjected to IF at −20, −30, and −40 °C, respectively (Table 2). The roundness of samples frozen by IF at −20, −30, and −40 °C was 0.71, 0.76, and 0.84 times higher, respectively, than that frozen by AF (Table 2), with the roundness of samples produced by IF at −40 °C found to be 44.83% greater than that produced by AF. This showed that a higher freezing rate was conducive for maintaining microstructural integrity. IF at −40 °C appeared to afford the smallest ice crystals (Figure 2e), which was mainly attributed to it having the shortest zone of maximum ice crystallization (Table 1), immediately removing latent heat from the samples, and having a lower nucleation temperature [25,27]. As a result, damage to the tissues and microstructure was reduced and the texture was generally better.

### 3.3. FT-IR Analysis

**Figure 3:**
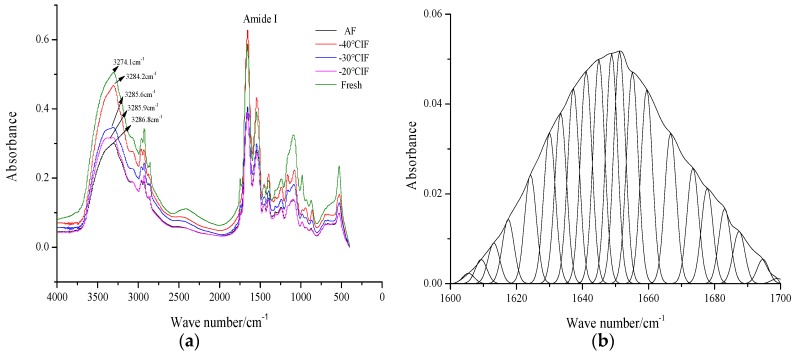
Fourier transform infrared spectroscopy (FT-IR) spectra of snakehead blocks treated under different freezing conditions: (**a**) FT-IR spectrum of myofibrillar protein; (**b**) deconvoluted infrared amide I band of the fresh sample.

In recent years, FT-IR spectroscopy has undergone rapid development to become a notable technology for the general analysis of secondary structure in proteins and polypeptides [32,33]. FT-IR spectra of snakehead MP treated under different freezing conditions are shown in Figure 3. Several typical protein bands were observed in the FT-IR spectra, including amide A (mainly H-bonding), amide B (C–H stretch), amide I (80% C=O stretch, 10% C–N stretch), amide II (60% N–H bend, 30% C–N stretch, and 10% C–C stretch), and amide III (complex bands resulting from several coordinate displacements) [34].

The samples showed strong and broad bands near 3300 cm-1, attributed to amide A. As shown in Figure 3, the amide A band appeared at 3274.1, 3286.8, 3285.9, 3285.6, and 3284.2 cm^−1^ for fresh, AF-treated, and IF-treated (−20, −30, and −40 °C) samples, respectively. Compared to the fresh samples, the amide A band, attributed to the stretching vibrations of intermolecularly H-bonded N–H and O–H bonds, was shifted to higher wavenumbers in all frozen samples. This was due to H-bonds that exist between protein molecules, which can shift the amide A band to lower wavenumbers, being interrupted by ice crystal formation during freezing [35]. Consequently, the amide A wavenumbers resulting from IF at −40 °C were lower than those of other treatments, mainly due to the high freezing rate and the formation of smaller and uniformly distributed ice crystals, which resulted in less disruption to the H-bonding system.

The amide I band, ranging from 1700 to 1600 cm^−1^ and mainly arising from C=O stretching vibrations, is the most significant band for the analysis of protein secondary structure and has been generally employed to monitor protein secondary structure conformations under various conditions [36]. The protein secondary structure is closely related to the vibration frequency of the amide I component. As shown in Figure 3a, the amide I band appeared at 1656.9 cm^−1^ in AF-treated samples and 1648.5, 1645.1, and 1641.9 cm^−1^ in IF samples treated at −20, −30, and −40 °C, respectively, while that of the fresh sample appeared at 1636.4 cm^−1^. Therefore, the amide I band was shifted to a higher wavenumber in all frozen samples, among which the lowest wavenumber was observed for IF treatment at −40 °C. This was attributed to the higher freezing rate, which prevented water diffusion in samples and resulted in the formation of small ice crystals and, consequently, less protein damage.

Figure 3b shows the fitting curve of the Fourier deconvoluted amide I band for MP composites. The various peaks and their frequencies correspond to β-sheets (1615–1637 cm^−1^ and 1682–1700 cm^−1^), α-helices (1646–1664 cm^−1^), random coils (1637–1645 cm^−1^), and β-turns (1665–1681 cm^−1^) [37,38].

Table 3 summarizes the relative percentage areas of the amide I band in the Fourier deconvoluted spectra of samples. These results show that the major components of the fresh sample were α-helices and β-sheets, while the minor components were β-turns and random coils. After the freeze–thawing process, the peak area percentage for α-helices in samples frozen under IF treatment at −20, −30, and −40 °C was 39.4 ± 1.13, 40.1 ± 1.26 and 42.7 ± 0.99, respectively, compared to 35.9 ± 1.27 under AF treatment at −20 °C. Furthermore, the peak area for random coils slightly increased. The H-bonding system that stabilized the protein structure was interrupted due to ice crystal formation, with hydrophobic and hydrophilic regions of proteins exposed to a new environment. Meanwhile, new intermolecular cross-links were formed within the protein molecules or between neighboring molecules [39].

### 3.4. Protein Thermal Stability Properties Determined by DSC

**Figure 4:**
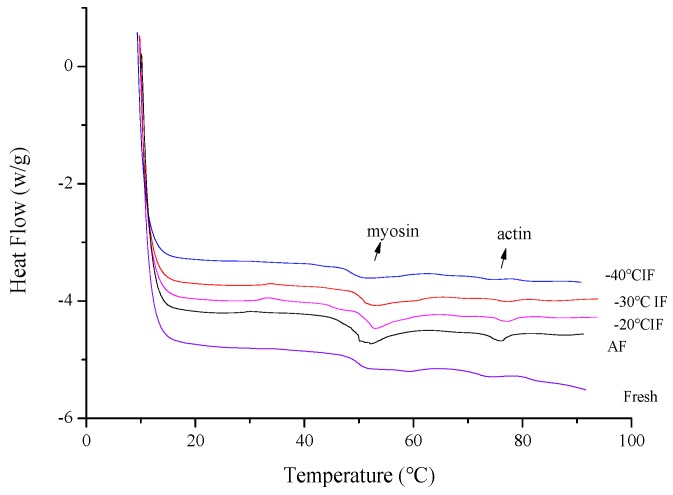
Changes in differential scanning calorimetry (DSC) scanning curve of snakehead muscle under different freezing conditions.

Differential scanning calorimetry (DSC) has been widely used to study the thermal stability of proteins [23,40]. After the freeze–thawing process, changes in the DSC thermograms reflected changes in the protein molecular structure, illustrating that the protein thermal stability had changed [41,42]. The DSC thermograms of snakehead muscle treated with different freezing conditions are shown in Figure 4. Thermograms for snakehead frozen by both IF and AF indicated two main transition peaks, namely peak 1 for myosin and peak 2 for actin. In agreement with rheological changes (Figure 4), DSC thermograms of myosin and actin resulted in endothermic peaks near 50 °C and 75 °C, respectively. For fresh samples, the myosin peak was relatively flat and the area under the DSC transition curve was small, indicating a higher thermal stability. The myosin peak of samples frozen by AF showed a significant variation, becoming broader and sharper than that resulting from IF. The change in shape of the myosin peaks suggested a lower cooperativity in the thermal denaturation of protein and indicated that AF caused more damage to the protein structure, leading to a decrease in protein thermal stability. The formation of ice crystals during the freezing processes affected the protein secondary structure and led to a lower thermal stability [43]. The differences in the DSC thermograms of snakehead muscle frozen under different conditions was reflected in changes in the denaturation maximum temperature (*T_max_*) and denaturation enthalpy (*∆H*) of the samples (Table 4). Changes in enthalpy values and transition temperatures can directly reflect protein denaturation, with higher *T_max_* and *∆H* values representing better protein structures [44]. After freezing, the *∆H* values were 1.43 ± 0.02, 1.55 ± 0.04, 1.58 ± 0.03, 1.61 ± 0.04 for myosin and 0.51 ± 0.02, 0.52 ± 0.01, 0.53 ± 0.02, 0.52 ± 0.01 for actin for samples treated by AF and IF at −20, −30, and −40 °C, respectively. Otherwise, the actin denaturation enthalpy did not show significant changes among the frozen and fresh samples (Table 4). This illustrated that the degree of actin denaturation caused by freezing was less than that of myosin. The DSC results confirm that the IF process reduced damage to the protein structure, which was beneficial for maintaining thermal stability.

## 4. Conclusions

The effects of IF at different temperatures (−20, −30, and −40 °C) and AF on the ice crystal size and protein properties of snakehead samples were investigated in this study. The results show that different treatments had a significant (*p* < 0.05) effect on the total freezing time. The cross-sectional area of ice crystals in IF-treated samples was considerably lower than that of AF-treated samples. The FT-IR and DSC results show that IF treatment better preserved the protein secondary structure and afforded a higher protein thermal stability compared to AF. Among the three IF treatments, the higher freezing rate played the most important role in guaranteeing the high frozen food quality, owing to the smaller size and increased roundness of the ice crystals, which resulted in less damage to the food microstructure. The results obtained herein improve one’s understanding of the IF process, with further investigation required to apply this knowledge to different food materials, such as fruits and vegetables, and compare this technique to other rapid-freezing technologies.

## Figures and Tables

**Table 1:** Freezing rate and freezing time distribution for each freezing stage under different conditions.

Sample	Pre-Cooling Stage (s) (4 to 0 °C)	Phase Change Stage (s) (0 to −5 °C)	Sub-Cooling Stage (s) (−5 to −18 °C)	Total Freezing Time (s)	Rate v/(cm·h^−1^)
AF	960 ± 82 ^a^	3412 ± 108 ^a^	2350 ± 114 ^a^	6722 ± 260 ^a^	0.38 ± 0.02 ^d^
−20 °C IF	102 ± 11 ^b^	310 ± 16 ^b^	266 ± 22 ^b^	678 ± 34 ^b^	3.42 ± 0.42 ^c^
−30 °C IF	58 ± 7 ^c^	226 ± 18 ^c^	108 ± 12 ^c^	392 ± 20 ^c^	5.63 ± 0.81 ^b^
−40 °C IF	51 ± 8 ^c^	125 ± 4 ^d^	90 ± 4 ^c^	266 ± 14 ^d^	8.65 ± 0.65 ^a^

The results are means ± standard deviation (*n* = 6). Values with different lowercase letters in the same column are significantly different (*p* < 0.05).

**Table 2:** Microscopic analysis of ice crystals in samples frozen by conventional air freezing (AF) and immersion freezing (IF) at −20, −30, and −40 °C.

Sample	Cross-Sectional Area (μm^2^)	Equivalent Diameter (μm)	Roundness
−20 °C AF	939.6 ± 134.2 ^a^	35.0 ± 3.3 ^a^	0.58 ± 0.03 ^c^
−20 °C IF	408.8 ± 76.8 ^b^	22.8 ± 2.0 ^b^	0.71 ± 0.02 ^b^
−30 °C IF	242.4 ± 47.0 ^c^	17.6 ± 1.2 ^c^	0.76 ± 0.03 ^b^
−40 °C IF	76.5 ± 6.4 ^d^	9.9 ± 0.4 ^d^	0.84 ± 0.05 ^a^

The results are means ± standard deviation (*n* = 100). Values with different lowercase letters in the same column are significantly different (*p* < 0.05).

**Table 3:** Percentage area contribution of amide I band component peaks of fish samples under different freezing conditions.

Sample	Peak Area of Amide I Band’s Component Peaks (%)			
	β-Sheet	Random Coil	α-Helix	β-Turn
Fresh	38.7 ± 2.97 ^a^	6.1 ± 0.56 ^b^	44.7 ± 2.69 ^a^	10.5 ± 0.42 ^b^
AF (−20 °C)	30.3 ± 1.56 ^b^	18.5 ± 1.56 ^a^	35.9 ± 1.27 ^d^	15.3 ± 0.71 ^a^
−20 °C IF	31.4 ± 2.55 ^b^	14.9 ± 1.27 ^c^	39.4 ± 1.13 ^bc^	14.3 ± 0.85 ^ac^
−30 °C IF	31.2 ± 2.69 ^b^	14.6 ± 0.57 ^c^	40.1 ± 1.26 ^b^	14.1 ± 0.42 ^ac^
−40 °C IF	32.6 ± 3.11 ^ab^	11.5 ± 0.99 ^d^	42.7 ± 0.99 ^a^	13.2 ± 0.99 ^c^

The results are means ± standard deviation (*n* = 6). Values with different lowercase letters in the same column are significantly different (*p* < 0.05).

**Table 4:** DSC analysis results for different freezing processes.

Sample	Myosin		Actin	
	T_max_ (°C)	∆H (J/g)	T_max_ (°C)	∆H (J/g)
Fresh	52.67 ± 0.23 ^a^	1.75 ± 0.04 ^a^	75.63 ± 0.32 ^a^	0.53 ± 0.01 ^a^
AF (−20 °C)	51.05 ± 0.27 ^b^	1.43 ± 0.02 ^c^	74.06 ± 0.22 ^b^	0.51 ± 0.02 ^a^
−20 °C IF	51.52 ± 0.49 ^ab^	1.55 ± 0.04 ^b^	74.59 ± 0.24 ^ab^	0.52 ± 0.01 ^a^
−30 °C IF	51.70 ± 0.37 ^ab^	1.58 ± 0.03 ^b^	74.94 ± 0.44 ^ab^	0.53 ± 0.02 ^a^
−40 °C IF	52.25 ± 0.62 ^ab^	1.61 ± 0.04 ^b^	75.44 ± 0.28 ^ab^	0.52 ± 0.01 ^a^

Values with different lowercase letters in the same column are significantly different (*p* < 0.05).
